# Natural Compound from Olive Oil Inhibits S100A9 Amyloid Formation and Cytotoxicity: Implications for Preventing Alzheimer’s Disease

**DOI:** 10.1021/acschemneuro.0c00828

**Published:** 2021-05-12

**Authors:** Manuela Leri, Himanshu Chaudhary, Igor A. Iashchishyn, Jonathan Pansieri, Željko M. Svedružić, Silvia Gómez Alcalde, Greta Musteikyte, Vytautas Smirnovas, Massimo Stefani, Monica Bucciantini, Ludmilla A. Morozova-Roche

**Affiliations:** † Department of Experimental and Clinical Biomedical Sciences “Mario Serio”, University of Florence, 50134 Florence, Italy; § Department of Neuroscience, Psychology, Drug Research and Child Health, University of Florence, 50139 Florence, Italy; ⊥ Department of Medical Biochemistry and Biophysics, Umeå University, 90187 Umeå, Sweden; ¶ Department of Biotechnology, University of Rijeka, HR 51000 Rijeka, Croatia; ∇ Institute of Biotechnology, Life Sciences Center, Vilnius University, LT-10257 Vilnius, Lithuania

**Keywords:** amyloid, cytotoxicity, neurodegeneration, oleuropein aglycone, plant polyphenols, S100A9

## Abstract

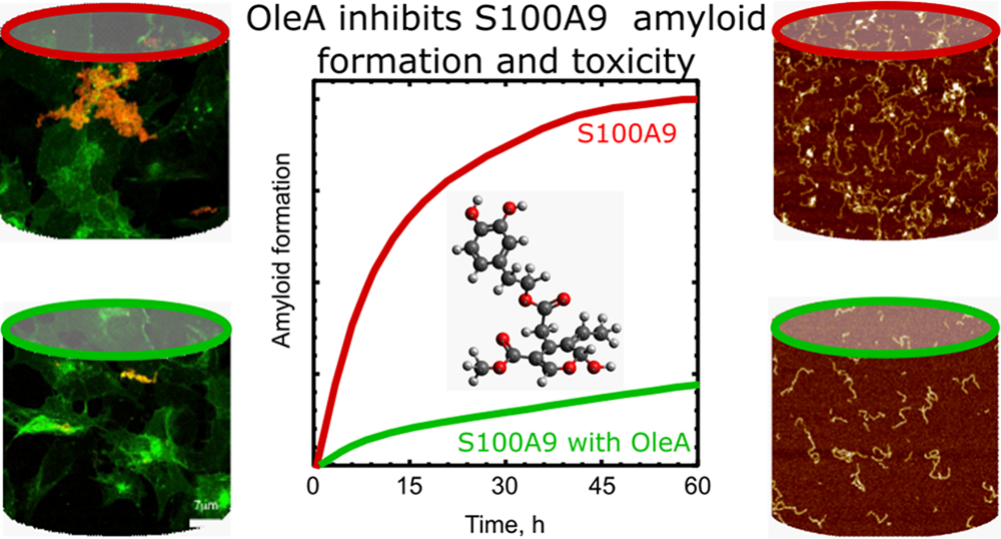

Polyphenolic compounds in the Mediterranean diet have received increasing attention due to their protective properties in amyloid neurodegenerative and many other diseases. Here, we have demonstrated for the first time that polyphenol oleuropein aglycone (OleA), which is the most abundant compound in olive oil, has multiple potencies for the inhibition of amyloid self-assembly of pro-inflammatory protein S100A9 and the mitigation of the damaging effect of its amyloids on neuroblastoma SH-SY5Y cells. OleA directly interacts with both native and fibrillar S100A9 as shown by intrinsic fluorescence and molecular dynamic simulation. OleA prevents S100A9 amyloid oligomerization as shown using amyloid oligomer-specific antibodies and cross-β-sheet formation detected by circular dichroism. It decreases the length of amyloid fibrils measured by atomic force microscopy (AFM) as well as reduces the effective rate of amyloid growth and the overall amyloid load as derived from the kinetic analysis of amyloid formation. OleA disintegrates already preformed fibrils of S100A9, converting them into nonfibrillar and nontoxic aggregates as revealed by amyloid thioflavin-T dye binding, AFM, and cytotoxicity assays. At the cellular level, OleA targets S100A9 amyloids already at the membranes as shown by immunofluorescence and fluorescence resonance energy transfer, significantly reducing the amyloid accumulation in GM1 ganglioside containing membrane rafts. OleA increases overall cell viability when neuroblastoma cells are subjected to the amyloid load and alleviates amyloid-induced intracellular rise of reactive oxidative species and free Ca^2+^. Since S100A9 is both a pro-inflammatory and amyloidogenic protein, OleA may effectively mitigate the pathological consequences of the S100A9-dependent amyloid-neuroinflammatory cascade as well as provide protection from neurodegeneration, if used within the Mediterranean diet as a potential preventive measure.

## Introduction

Alzheimer’s disease (AD) and Parkinson’s disease (PD) as well as other amyloid-related neurodegenerative ailments are age-related multifaceted pathological conditions in which a broad range of events lead to the impairment of brain activity with cognitive disability as the main clinical hallmark. The majority of nonfamilial neurodegenerative diseases are characterized by gradual development, usually spanning many years, and with the appearance of their clinical signs at the later stage, when cell decay is obvious and cannot be reversed. In spite of numerous efforts to understand the specific molecular and cellular mechanisms underlying AD and PD pathologies and their potential triggers, effective disease-modifying treatments still remain elusive. Therefore, the delay of the disease onset by a healthy lifestyle and diet remains the best strategy.

Neuroinflammation and amyloid burden are two characteristic pathologies of AD, PD, and other neurodegenerative disorders.^1−3^ The amyloid cascade is linked to the failure of polypeptides to adopt and retain their native structure and function, leading to their conversion into toxic amyloid oligomers and insoluble amyloid deposits. The amyloid conversion impairs the physiological function of native polypeptide and leads to a gain in amyloid cytotoxicity. The latter further interferes with signaling pathways and cell homeostasis, including redox equilibrium, free Ca^2+^ level, and proteostasis.^4^ Distinct polypeptides are associated with particular human disorders, notably the Aβ peptide with AD and α-synuclein with PD; however, the growth and deposition of amyloids are shared features of numerous amyloid neurodegenerative diseases.^2^ Previously, neuroinflammation was viewed as a reaction to the degeneration process; however, presently, it is known that neuroinflammation may play a central role in triggering and promoting neurodegeneration, since inflammation processes in the brain environment may promote amyloid self-assembly. In AD and PD, the affected tissues in the brain display a range of damaging responses, including substantial activation of microglia and an increase in the level of inflammatory cytokines, which sustain inflammation and exacerbate neurodegeneration.^5−8^

Recently, we have reported that the pro-inflammatory and amyloidogenic protein S100A9 is a major contributor to both the amyloid and neuroinflammatory cascades in AD, PD, and traumatic brain injury; importantly, traumatic brain injury may play a role of the precursor state for numerous neurodegenerative conditions. Thus, S100A9 may bridge the amyloid self-assembly and inflammation into the distinctive and common neurodegenerative disease feature: the amyloid-neuroinflammatory cascade.^9−11^ S100A9 has been described as an alarmin implicated in numerous signaling pathways in cancers and inflammation-related diseases.^12,13^ The high level of S100A9 expression was also observed in malaria,^14^ ischemia,^15^ obesity,^16^ and cardiovascular disease.^17^ The S100A9 mRNA was found to be abundant in numerous aged mammalian organs, also involving the central nervous system; consequently, it was suggested that an age-associated inflammation is implicated and persists due to continued S100A9 production.^18^

Previously, we have found that S100A9 is a very amyloidogenic protein; i.e., *in vitro* S100A9 aggregates easily under a physiological environment, and its self-assembly is well-defined by a nucleation-autocatalytic growth model.^19^ The S100A9 self-assembly into amyloids results in acquired cytotoxicity, exceeding the levels of Aβ peptide amyloid cytotoxicity in AD^11^ and α-synuclein amyloid cytotoxicity in PD.^10^ This implies that the increased level of S100A9 persisting during inflammation may provoke its amyloid self-assembly, deposition, and tissue damage, which we observed in neurodegenerative diseases,^10,11^ in the aged prostate,^20^ and in the cell model for S100A8 and S100A9 amyloid accumulation.^21^ Indeed, in AD, S100A9 coassembles with Aβ, leading to intracellular amyloid accumulation and extracellular amyloid plaque growth;^11^ in PD, S100A9 coaggregates with α-synuclein, which is manifested in Lewy body development,^10^ while in traumatic brain injury, S100A9 protein deposits into numerous precursor plaques.^9^ We have found also that S100A9 levels in CSF of AD and mild cognitive impairment match those of Aβ, further confirming its involvement in the amyloid-neuroinflammatory cascade even at the disease preclinical and mild stages.^22^ Thus, S100A9 may easily aggregate by itself or coaggregate with various other proteins and peptides involved in the amyloid-related diseases and may be considered as an object for therapeutic treatments. This is particularly important, since the whole amyloid cascade may be potentially affected or reversed by targeting the amyloid self-assembly of its single component, such as the S100A9 protein.

Despite significant efforts over the past decades, there are still no drugs available to modify amyloid neurodegenerative disease progression, and despite many compounds being characterized by promising amyloid inhibiting properties in *in vitro* experiments, they failed in the clinical trials. Many low molecular weight substances, peptides, and various antibodies were examined as prospective amyloid modifiers, yet potent, natural compounds without significant side effects are still needed for effective therapeutic treatments.

**Figure 1:**
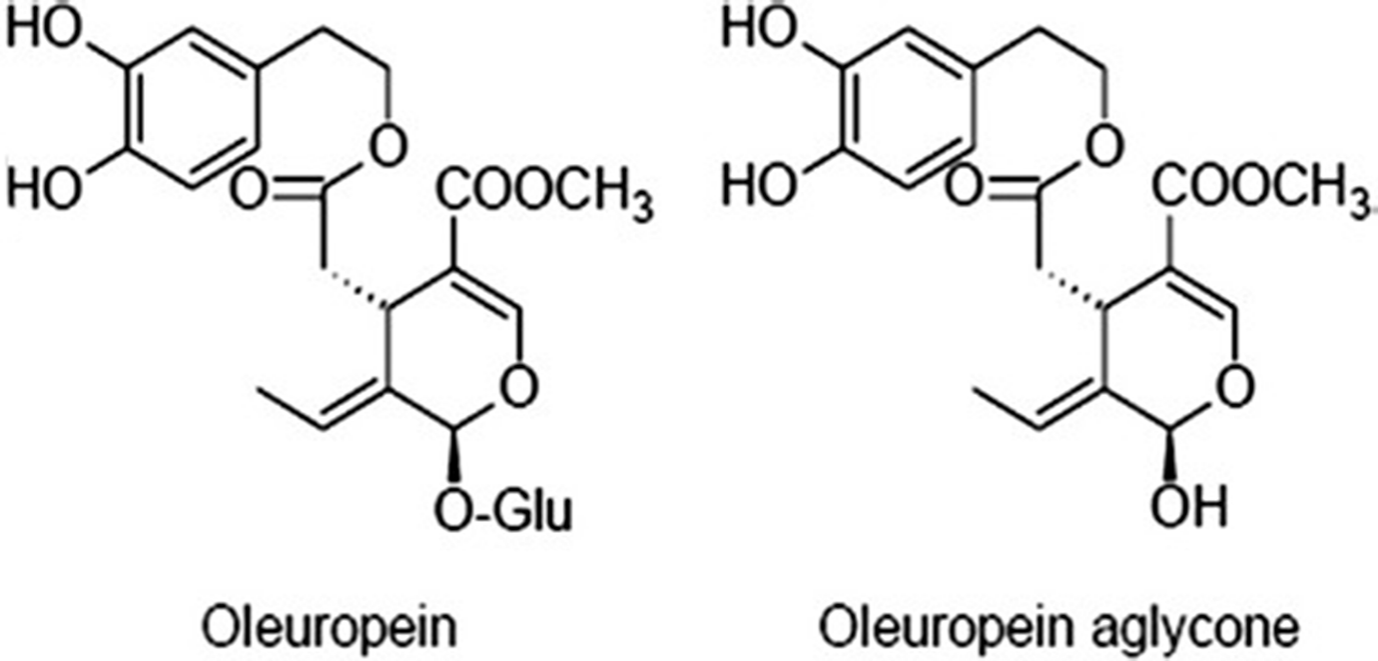
Schematic presentation of the chemical structure of oleuropein and oleuropein aglycon (OleA).

Numerous clinical trials and population analysis support the notion that the Mediterranean diet correlates with a decreased occurrence of age-related ailments, such as neurodegenerative disorders.^23−25^ The most important characteristic of the Mediterranean diet is the high consumption of natural plant phenols^24^ found in many plant products, such as extra virgin olive oil (EVOO). Oleuropein is a secoiridoid glycoside containing phenylpropanoid alcohol, which can be produced from the biosynthesis of mevalonic acid. After the maturation process and extraction of EVOO, as a result of the β-glucosidase enzyme activity, this secoiridoid is found in its aglyconic form as oleuropein aglycone (OleA) (Figure 1). Oleuropein and its derivatives found in EVOO received particular attention due to their multiple important and protective functionalities, including antioxidant, antiviral, antitumor, antimicrobial, cardioprotective, hepatoprotective, neuroprotective, antiaging, antidiabetic, and anti-inflammatory effects.^23^ Olive polyphenols, such as OleA and its main metabolite hydroxytyrosol, were studied for many years by us and other researchers regarding their effects on the aggregation pathways of various polypeptides involved in amyloid and neurodegenerative diseases, including Aβ peptide, tau protein, amylin, transthyretin, and α-synuclein.^24−31^ While multiple effects of olive polyphenols have been detected at the molecular, cellular, and animal model levels, the specific mechanisms remain elusive, and important questions still need to be addressed as to whether there is a generic target or numerous targets along multiple aggregation pathways, which could be individual for each particular polypeptide. In particular, the reduced toxicity of the amyloids developed in the olive polyphenol presence was demonstrated in both cellular and murine models. In cultured cells, their amyloid protection was very significant and started at the level of the cellular membrane, perturbing various signaling pathways.^32,33^ The experiments were carried out also on the TgCRND8 transgenic mouse model of plaque deposition; the mice were fed daily with an OleA or HT-integrated diet, and it was revealed that the animals displayed a strong improvement of the memory and behavioral deficits compared to untreated littermates.^34^ These were accompanied by a substantial decrease in the quantities of amyloid plaques and the level of neuroinflammation. Moreover, a strong activation of the autophagic response was detected^34^ with possible involvement of epigenetic modifications.^35^

Here, we studied the effect produced by OleA on S100A9 amyloid aggregation using a range of complementary methods in biophysics, biochemistry, cell biology, and advanced microscopy. We demonstrated how OleA mitigates S100A9 amyloid cytotoxicity in the human neuroblastoma SH-SY5Y cell line. Altogether, these findings shed light on the molecular and cellular determinants of OleA protection from toxic amyloids, highlighting the potential of olive polyphenols in averting AD and other neurodegenerative diseases associated with the amyloid-neuroinflammatory cascade.

## Results and Discussion

### Inhibiting S100A9 Amyloid Aggregation by OleA

**Figure 2:**
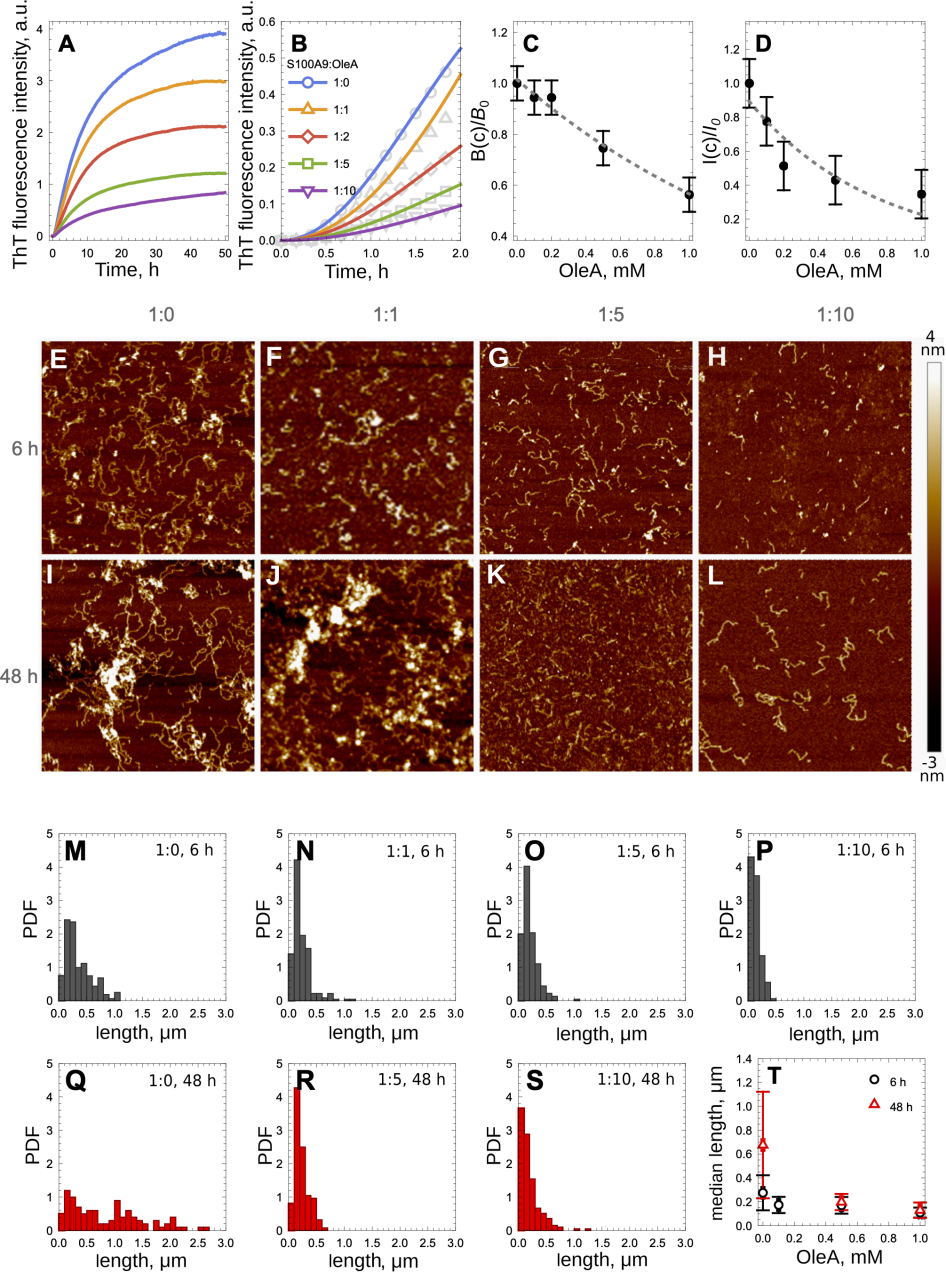
Inhibition of S100A9 amyloid formation in the presence of increasing OleA concentrations observed by the ThT fluorescence assay and AFM. (A) S100A9 amyloid kinetics in the presence of increasing OleA concentrations monitored by the ThT assay. (B) Fitting of the initial parts of S100A9 amyloid kinetics with the nucleation-dependent polymerization model. (C) Ratios of the effective rate constants of S100A9 amyloid kinetics in the presence and absence of OleA (*B*_0_ is the effective rate constant in the absence of OleA, and *B*(*c*) is the rate constant at an OleA concentration of *c*). (D) Ratios of plateau intensities of S100A9 amyloid kinetics in the presence and absence of OleA (*I*_0_ is the plateau intensity in the absence of OleA, and *I*(*c*) is the plateau intensity at an OleA concentration of *c*). AFM images of S100A9 amyloid species without OleA and with increasing OleA concentrations after a 6 h (E–H) and 48 h (I–L) incubation, respectively. Amyloid fibril length distributions without OleA and with increasing OleA concentrations after a 6 h (M–P) and 48 h (Q–S) incubation, respectively, measured in AFM images. (T) Median length of S100A9 amyloid fibrils after a 6 h (black circles) and 48 h (red triangles) incubation, respectively, with increasing OleA concentrations. Molar ratios of S100A9 to OleA and the time of incubation are indicated in the figures. 100 μM S100A9, PBS, pH 7.4, and 42 °C. *z*-scale in the AFM images is indicated on the right with the bar color gradient from dark brown to light yellow. *x*,*y*-AFM scan sizes are 2 × 2 μm.

The kinetics of S100A9 amyloid formation in the presence of increasing OleA concentrations were carried out using the thioflavin-T (ThT) fluorescence assay as described previously^36^ and shown in Figure 2A. S100A9 alone assembles into amyloid structures by the nucleation-dependent polymerization mechanism,^19,37^ and its kinetics are characterized by the lack of a significant lag phase and steep growth phase, prior to reaching the plateau level. Upon an increase in the OleA concentrations, the slopes of amyloid growth, reflecting the kinetics rates, as well as the plateau levels where the amyloid assembly reached semiequilibrium noticeably decreased.OleA incubated alone under the same conditions did not interact with ThT as shown in Figure S1 and therefore did not contribute to the overall ThT signal in the corresponding mixtures. The initial parts of the S100A9 amyloid kinetic traces, corresponding to <20% maximal ThT fluorescence intensity values, were fitted using the nucleation-dependent polymerization model^38^ (Figure 2B). The effective amyloid growth rate constants as well as the plateau levels were significantly decreased upon the increase in OleA concentrations (Figure 2C,D), reflecting the decrease in the overall amount of self-assembled amyloids.

To monitor amyloid morphology during the amyloid self-assembly process, the atomic force microscopy (AFM) imaging was performed after a 6 and 48 h incubation of S100A9 samples in the presence and absence of OleA concentrations as demonstrated in Figure 2E–L. Already after a 6 h incubation, S100A9 alone formed flexible fibrils with ca. 2 nm height in the AFM cross sections and 0.28 ± 0.15 μm median length (Figure 2E,M). After a 48 h incubation, S100A9 fibrils grew in length, showing a broad distribution with 0.7 ± 0.4 μm median length, and collapsed and clumped aggregates were formed (Figure 2I,Q). Indeed, it has been demonstrated previously that S100A9 flexible fibrils tend to clump upon prolonged incubation.^39^ Upon incubation with increasing OleA concentrations, the length and relative quantity of S100A9 amyloid fibrils captured by AFM imaging decreased after both 6 and 48 h incubation periods as demonstrated in Figure 2T. Importantly, the fibrils do not grow further upon increasing the incubation time from 6 to 48 h as summarized by presenting the median values of fibrillar length at different OleA concentrations in Figure 2T. The distributions of fibrillar length in the presence of OleA becomes significantly narrower compared to S100A9 alone (Figure 2M–S). The AFM heights of S100A9 fibrils in all samples remain about the same, ca. 2 nm, in the AFM cross sections. Interestingly, the decrease in fibrillar length in the presence of OleA correlates with the reduction of total amount of amyloids detected by ThT binding (Figure 2D,T).

The dot blot analysis indicates that the interaction of S100A9 aggregated samples with generic antiamyloid oligomer specific A11 antibodies is noticeable after a 6 h incubation and subsides upon an increase in the OleA concentration, being completely abolished at a S100A9 to OleA molar ratio of 1:10 (Figure S2). After a 48 h incubation, the amount of oligomeric species was significantly diminished in the S100A9 amyloid sample in the absence of OleA and completely absent in the presence of all concentrations of OleA.

**Figure 3:**
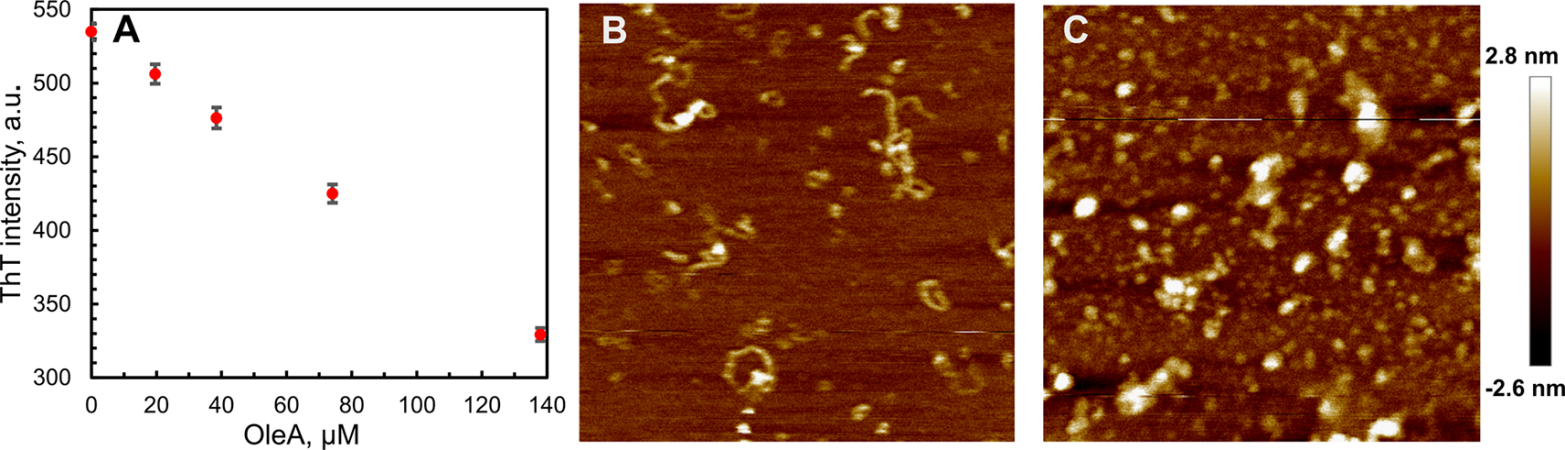
Preformed S100A9 fibrils disintegrated by OleA. (A) Decrease of ThT dye fluorescence upon the addition of OleA to S100A9 fibrils formed after a 48 h incubation. (B) AFM images of S100A9 fibrils without OleA. (C) AFM image of S100A9 aggregates after the addition of 137 μM OleA to the sample shown in (B). 75 μM S100A9 was subjected to fibrillation in PBS, pH 7.4, at 42 °C. Scan sizes are 2.5 × 2.5 μm.

Interestingly, upon the addition of OleA to preformed S100A9 fibrils assembled during the 48 h incubation, we observed the decrease of ThT fluorescence, indicating the decrease of amyloid quantity in these samples (Figure 3A). AFM imaging demonstrated that flexible fibrils of S100A9 observed prior to the addition of OleA (Figure 3B) have collapsed into nonfibrillar aggregates after the addition of OleA, i.e., at a 1:2 molar ratio of S100A9 to OleA (Figure 3C).

**Figure 4:**
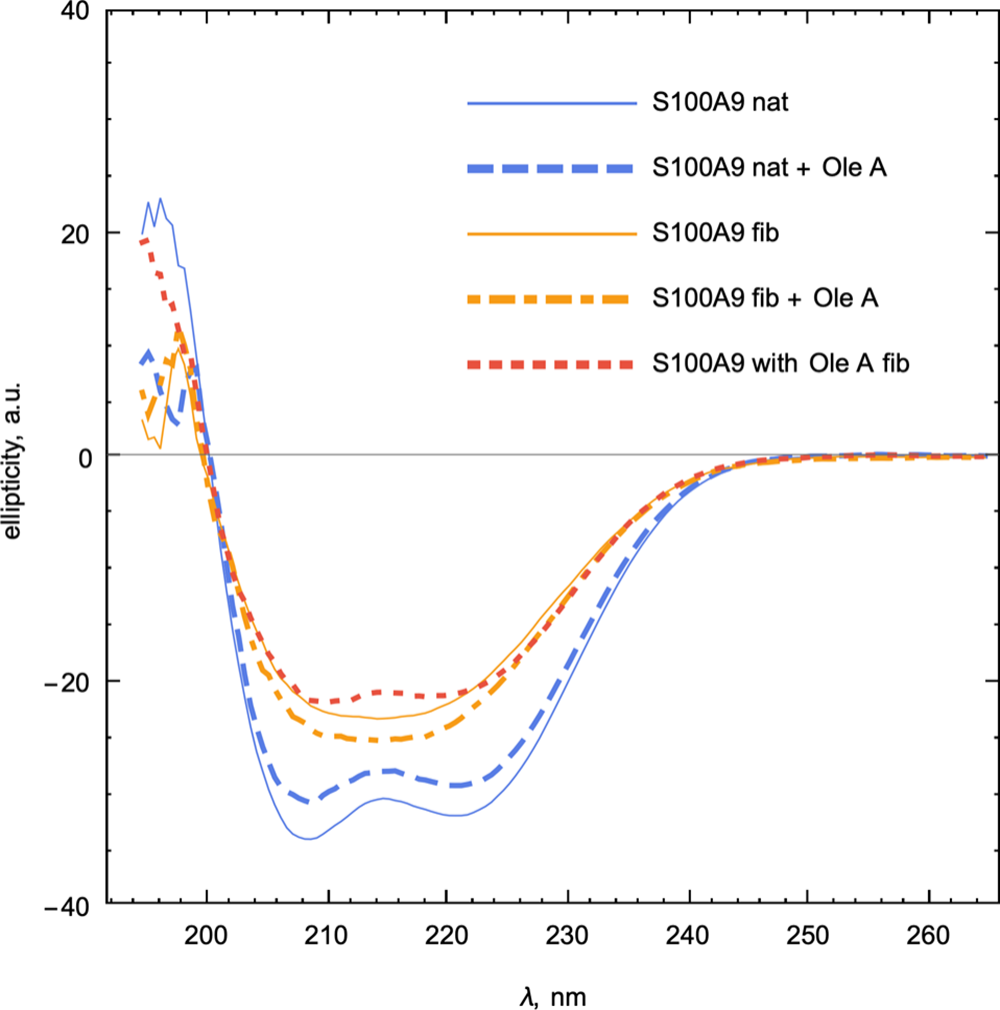
Far-UV CD spectra of native and amyloid S100A9 and the OleA effect on them. Spectra were recorded at 25 °C at the following conditions: 75 μM S100A9 in all samples and 1:2 S100A9 to OleA molar ratio, if OleA is added. PBS, pH 7.4. S100A9 fibrils were formed during the 48 h incubation at 42 °C. Labeling of the samples is shown in the legend.

The far-UV circular dichroism (CD) spectrum of native S100A9 is characteristic for α-helical protein, displaying minima at 222 and 208 nm, and it was not perturbed upon the addition of OleA (Figure 4). This indicates that OleA perturbs the S100A9 structure locally rather than globally. The spectrum of fibrillated S100A9 displayed some changes in its shape with a broad minimum of ellipticity at 214–216 nm, reflecting the development of the β-sheet. It is not excluded that some α-helical structure was preserved in the S100A9 fibrils, since the spectrum is broader then the typical β-sheet spectrum; i.e., the remaining α-helices still can be packed at the fibrillar interface. When S100A9 was incubated in the presence of OleA to induce its amyloid formation, the shape of the CD spectrum remained close to the native-like one, demonstrating that OleA indeed inhibits amyloid development. Interestingly, when OleA was added to the preformed fibrils of S100A9, it induced the changes in the far-UV CD spectrum, which resembles a mixture of α-helices and β-sheets. This indicates that some reduction of the β-sheet content took place (Figure 4), which corresponds to the nonfibrillar aggregate formation described above (Figure 3).

Thus, all above experiments demonstrate consistently that OleA produces a significant inhibiting effect on S100A9 amyloid formation by inhibiting its amyloid oligomerization (dot blot analysis with A11 antibodies) and cross-β-sheet formation (far-UV CD), reducing the effective rate of amyloid assembly (kinetic assay), reducing the length of amyloid fibrils (AFM), and thus diminishing the overall quantity of the amyloids (ThT fluorescence and AFM). Moreover, OleA is able to convert the already preformed S100A9 fibrils into unstructured aggregates and potentially make them more susceptible to the clearance process in the body.

### Interaction of OleA with Native and Amyloid S100A9 followed by Intrinsic Fluorescence

**Figure 5:**
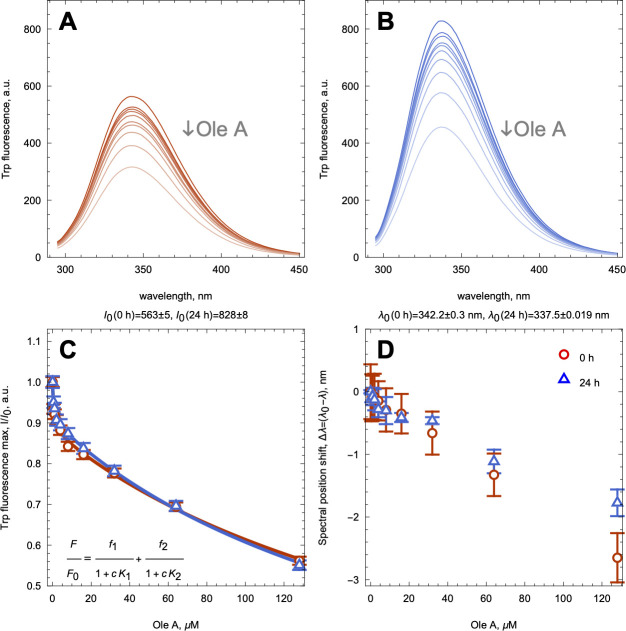
Interaction of native and amyloid S100A9 with OleA monitored by intrinsic fluorescence. Fluorescence spectra of native (A) and amyloid (B) S100A9 upon increasing concentrations of OleA. (C) Relative fluorescence intensities at the spectral maximum of native (blue triangles) and amyloid (red circles) S100A9 upon increasing OleA concentrations. Fitting curves are shown by solid lines in the corresponding color. The two binding site model is shown in the inset. (D) Shifts of fluorescence spectral maxima of native and amyloid S100A9 determined by the first derivative method upon increasing OleA concentrations shown in the same symbols as in (C). Four μM S100A9, PBS, pH 7.4, and 42 °C.

To shed light on OleA interactions with native and amyloid S100A9, we have titrated both samples with increasing concentrations of OleA and followed the changes by the intrinsic fluorescence of Trp 88. For the titration, we used the S100A9 amyloid sample incubated for 24 h at a 100 μM concentration and subsequently diluted it to 4 μM to prevent fibrillar clumping (Figure 2), and native S100A9 was also taken at 4 μM (both in monomer equivalent). In both samples, we have observed a decrease of the intrinsic fluorescence intensities upon an increase in the OleA concentrations, demonstrating nonhyperbolic concentration dependences, which were fitted with a two binding site model as shown in Figure 5A–C. Both fitting curves were largely overlapped, suggesting the same binding sites in the native and amyloid S100A9 species. Since OleA itself can associate into dimer, trimer, and larger species^40^ and act as a fluorescence quencher of Trp fluorescence,^29^ the apparent dissociation constants (*K*_d_) of 0.5 and 200 μM may reflect the cumulative effect of binding and fluorescence quenching produced by the OleA polymorphic species.

Interestingly, native and amyloid S100A9 are characterized by different fluorescence maxima of 343 and 337 nm, respectively, indicating that within the fibrils Trp 88 is present in the more hydrophobic environment. The fluorescence spectral maxima of both native and amyloid S100A9 samples were shifted by ca. 3 nm toward the shorter wavelengths upon an increase in the OleA concentrations (Figure 5D), indicating that in both species Trp 88 becomes even more buried into the hydrophobic interior upon OleA binding.

### MD Simulation of the OleA Interaction with S100A9

**Figure 6:**
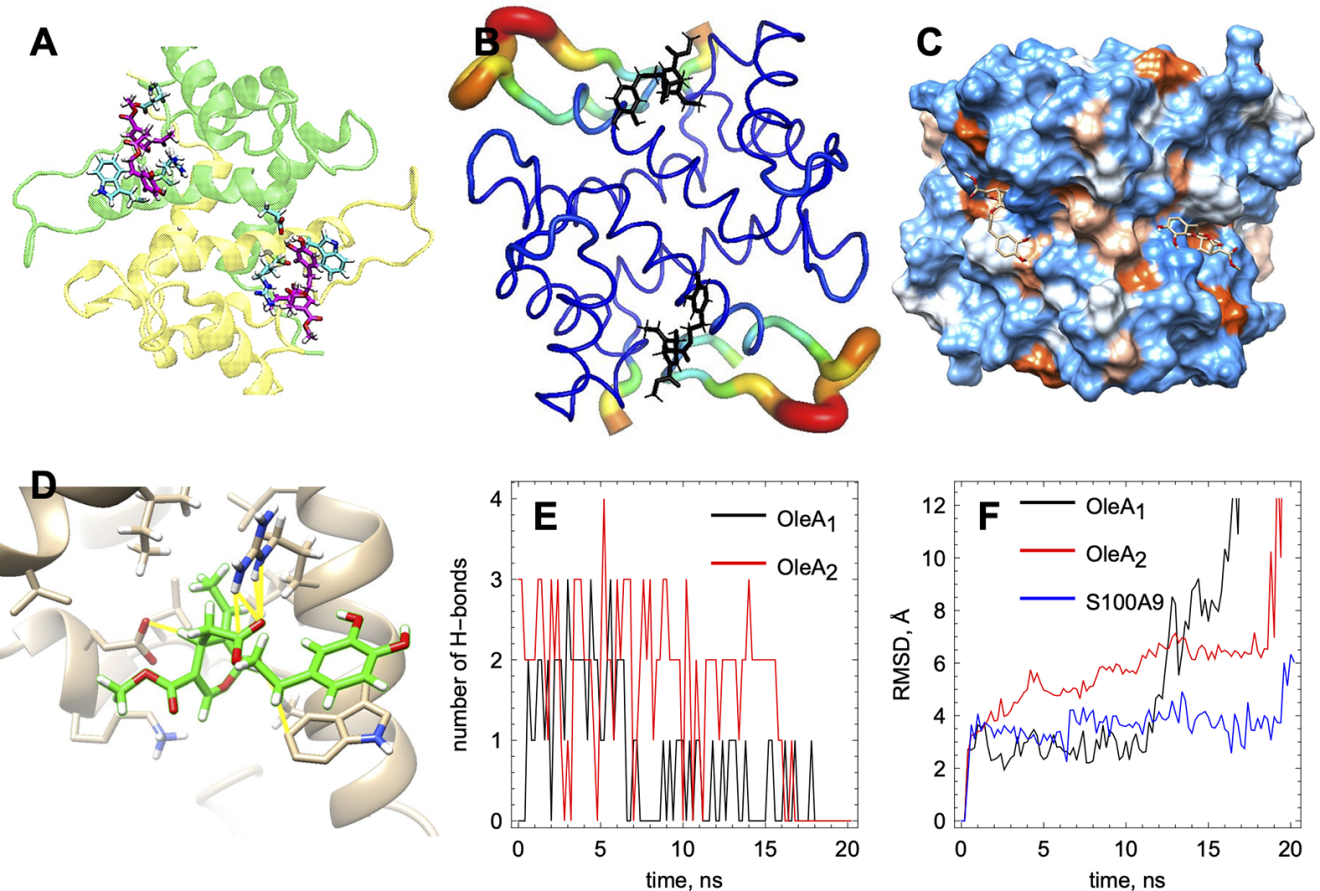
MD simulation of the interactions of native S100A9 with OleA. (A) Binding of one OleA molecule per subunit of S100A9 dimer. S100A9 monomers are shown by the ribbon diagram in yellow and green colors, respectively. The amino acid side chains of S100A9, which form binding interactions with OleA, are shown by cyan sticks. OleA molecules are shown by magenta sticks. OleA forms hydrogen bonds with Glu 52 and Arg 85 and π–π stacking interactions with Trp 88. (B) B-factor presentation of the S100A9 dimer backbone shows that OleA (in black sticks) binds to the protein site with low mobility (shown in blue). The loops with high mobility are shown by thicker tubes with a color gradient from yellow to red, corresponding to increasing mobility. (C) S100A9 dimer surface presentation in space filling and colors based on the polarity of the amino acid residues: hydrophilic residues, blue; hydrophobic residues, brown; residues with intermediate properties, white. OleA (shown by sticks) binds to a shallow hydrophobic cavity on the S100A9 surface. (D) Detailed presentation of the OleA orientation and binding interactions (presented in yellow) highlighting: π–π stacking between Trp 88 and OleA, hydrogen bonding between Arg 85 and the carbonyl oxygen of OleA, and the proximity between Glu 52 and the ester group of OleA. (E) Number of hydrogen bonds between each OleA molecule and S100A9 dimer. OleA molecules dissociate when the number of hydrogen bonds falls to zero. Hydrogen bonds were defined as the interactions between the polar groups with an angle of less than 20° and less than 3.0 Å distance. (F) Root mean square deviation (RMSD) values demonstrating the mobility of each OleA molecule and S100A9 dimer. Ligand dissociation results in the rapid increase in the RMSD values.

To shed further light on OleA interactions with S100A9, we have performed the MD simulation of the native S100A9 dimer in the presence of two molecules of monomeric OleA, taking into account that S100A9 self-assembles into a homodimer under native and also destabilizing conditions^11,37^ (Figure 6). The S100A9 dimer is characterized by a flat surface with no deep cavities, and molecular docking reveals only one major OleA binding site per subunit (Figure 6A). The B-factor presentation of the S100A9 molecules shows that OleA docks into the least mobile part of the S100A9 structure (Figure 6B).^41^ The space filling presentation of the S100A9 dimer with the hydrophobic and charged/hydrophilic residues indicates that OleA binds in a shallow hydrophobic cavity in each subunit (Figure 6C) by forming several dynamic H bonds (Figure 6D). Specifically, these are the hydrogen bonds between S100A9 Glu 72 and the OH group on the OleA aromatic ring, Glu 52, and methyl-ester end of OleA as well as Arg 85 and the carbonyl oxygen on OleA (Figure 6D). In addition, π–π stacking interactions between the Trp 88 and OleA aromatic ring are formed (Figure 6D). The number of dynamic hydrogen bonds per time frame between each OleA molecule and S100A9 subunit varies between 1 and 3 (Figure 6E), which together with π–π stacking interactions may ensure micromolar to submicromolar binding affinities, observed in the fluorescence titration measurements (Figure 5). The root mean square deviation (RMSD) graph demonstrates that, due to the lack of a well-defined binding cavity, OleA molecules may dissociate from the S100A9 dimer after ca. 15 ns (Figure 6F). The high mobility of OleA at the S100A9 surface can disrupt the optimal protein–ligand orientations and the maximal number of binding interactions, suggesting that OleA binding may involve multiple events. The high-affinity interactions reflect the optimal ligand–protein orientation and the maximal number of binding interactions, while the suboptimal orientation and lower number of binding interactions may contribute to the lower binding affinities, consistent with the fluorescence titration experiments (Figure 5). OleA binding to the S100A9 dimer does not perturb its α-helical secondary structure beyond the binding site, which is consistent with the CD measurements described above.

Thus, the combined fluorescence titration and MD simulation experiments demonstrate consistently that OleA indeed interacts with native S100A9. The interactions involve multiple binding events due to the lack of specific OleA binding cavities on the S100A9 molecule (Figure 4C) similar to its interactions with other previously studied amyloidogenic polypeptides.^24,26,29,32^ Moreover, OleA can self-assemble into a larger species,^40^ which may also affect its apparent binding affinity. These were further supported by the similar binding of OleA with both native and amyloid S100A9 (Figure 5C).

### OleA Alleviates S100A9 Induced Cytotoxicity Measured by the MTT Assay

The effect of OleA on the S100A9 cytotoxicity induced in the neuroblastoma SH-SY5Y cell line was studied by the 3-(4,5-dimethylthiazol-2-yl)-2,5-diphenyltetrazolium bromide (MTT) cytotoxicity assay. Initially, neuroblastoma cells were treated for 24 h with various concentrations of S100A9 alone in either native or 48 h aged amyloid form (Figure S3A). S100A9 amyloids were more cytotoxic, reducing the cell viability by ca. 40–50% at 20 to 40 μM concentrations; native S100A9 also reduced the cell viability by ca. 20–30% at similar concentrations (Figure S3A). Since S100A9 is an alarmin and pro-inflammatory protein, it can induce cellular toxicity also in its native state via activation of RAGE and TLR-4 receptors.^42,43^

**Figure 7:**
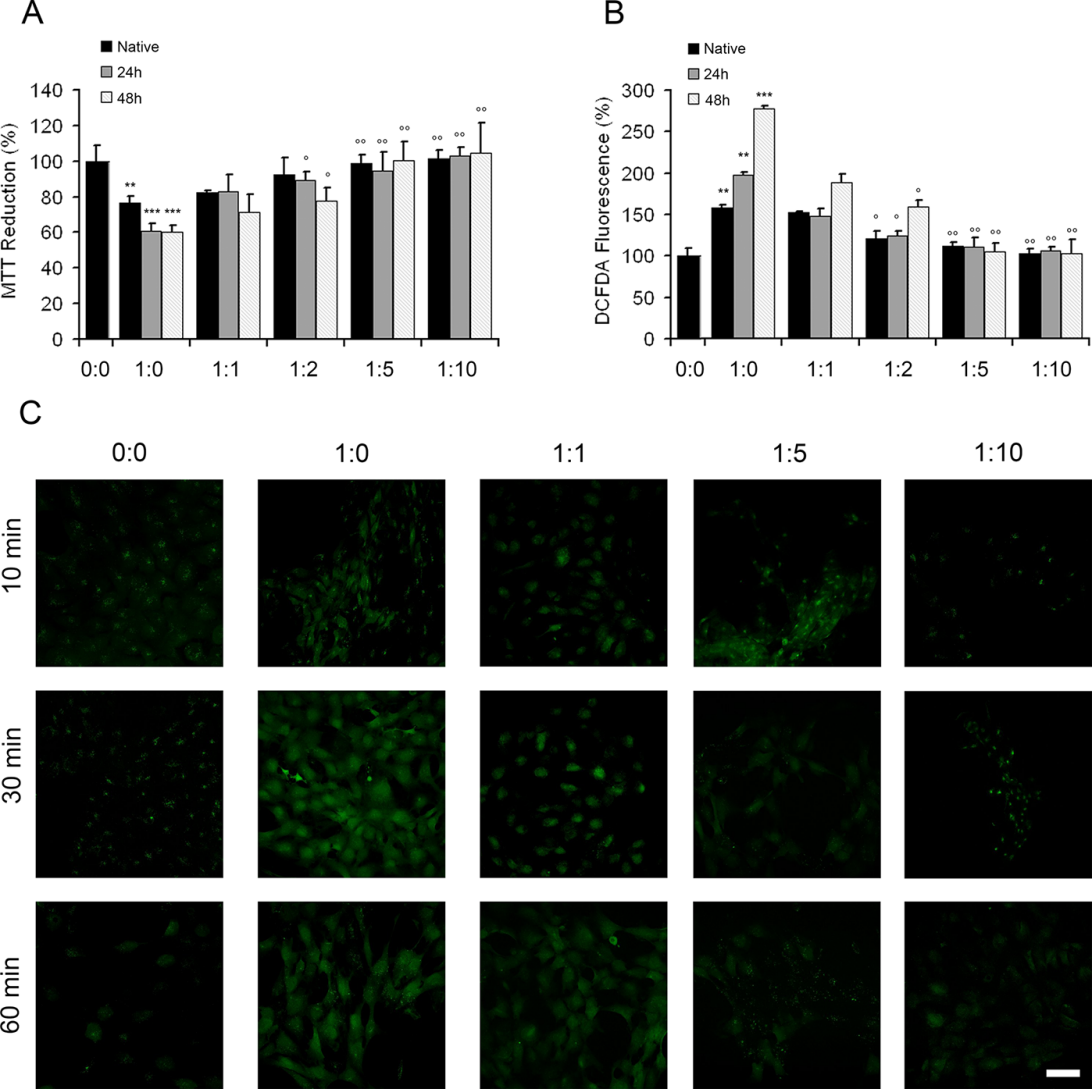
Cytotoxic effect of the S100A9 amyloids in the SH-SY5Y cells is mitigated by OleA. (A) Viability of SH-SY5Y cells measured by the MTT assay and (B) ROS levels in the SH-SY5Y cells measured by DCFDA fluorescence. Cells were exposed to 20 μM S100A9 samples for 24 h. S100A9 was added in either native or amyloid forms, which were incubated for 24 and 48 h in PBS, pH 7.4, at 42 °C, respectively, prior the addition to the cells. S100A9 to OleA molar ratios were 1:0, 1:1, 1:2, 1:5, and 1:10 as indicated along *x*-axis. The control corresponds to the untreated cells and is shown by a single black bar, where the absence of S100A9 and OleA is indicated as (0:0). The experimental bars corresponding to the addition of native S100A9 are shown in black, the addition of 24 h aged S100A9 amyloids are shown in gray, and the 48 h aged amyloids are shown in white. Error bars indicate the standard error of the mean of the independent experiments carried out in triplicate. ***p < 0.001 and **p < 0.01 versus control (0:0). °*p* < 0.05 and °°*p* < 0.01 versus cell viability in the presence of native or the corresponding aggregated S100A9 without OleA, i.e., molar ratio (1:0). (C) Confocal microscopy imaging of the intracellular free Ca^2+^ levels in the SH-SY5Y cells exposed for 10, 30, and 60 min to 20 μM S100A9 amyloids incubated for 48 h in the absence or in the presence of OleA at the molar ratios of S100A9 to OleA indicated in the figure. Scale bars are 14 μm in all images.

Then, SH-SY5Y cells were exposed for 24 h to 20 μM of either native or amyloid S100A9 samples aged for 24 and 48 h, respectively, under aggregating conditions (Figure 7A). All samples were prepared both without or with OleA at the following S100A9 to OleA molar ratios: 1:1, 1:2, 1:5, and 1:10 (in monomeric equivalent). Using the MTT test, we found that the S100A9 amyloids aged 24 and 48 h without OleA displayed the same cytotoxic effect, decreasing cell viability by about 40% (Figure 5A). Their cytotoxicity was mitigated, however, if S100A9 aggregates were incubated in the presence of OleA, then the cell viability was fully recovered at both 1:5 and 1:10 of S100A9 to OleA molar ratios (Figure 7A). Similar trend was observed for native S100A9, where the recovery of cytotoxicity correlated with the increasing concentrations of OleA, and full recovery was achieved at a 1:5 of S100A9 to OleA molar ratio.

The cytotoxicity of the preformed fibrils of S100A9 treated with OleA was also tested on the SH-SY5Y cells by the MTT assay. The results are shown in Figure S4. We observed a decrease of the cytotoxic effect of the preformed S100A9 amyloids when they were treated with OleA at a 1:2 molar ratio of S100A9 to OleA from 1 to 48 h. This data suggests that after 48 h of coincubation OleA disassembles already formed S100A9 fibrils into nonfibrillar aggregates as shown in Figure 2C, that are no longer toxic. Such an inhibitory effect was also observed after shorter treatment times, suggesting that OleA likely makes the fibrils harmless by solvating them before inducing their collapse into unstructured aggregates.

### OleA Mitigates the Perturbations in Reactive Oxidative Species (ROS) and Intracellular Ca^2+^ Levels Induced by S100A9 Amyloids

Free Ca^2+^ levels and reactive oxidative species imbalances are involved in the cellular toxicity of amyloid.^44,45^ Here, we examined whether OleA can act as a protective agent in these processes as well. Intracellular ROS levels were measured using the fluorescent probe CM-H_2_DCFDA (Figure 7B). In the cells exposed to native S100A9 as well as to the 24 and 48 h aged S100A9 amyloids, the ROS levels were increased by ca. 1.5-, 2.0-, and 2.8-fold, respectively, compared to untreated cells. Remarkably, the presence of OleA reduced ROS production in all cells exposed to both native and amyloid S100A9. The ROS level returned to the level of untreated cells at 1:5 and 1:10 ratios of S100A9 to OleA (Figure 7B).

We evaluated the changes of intracellular free Ca^2+^ levels in neuroblastoma cells treated with native S100A9 and S100A9 amyloids aged for 24 and 48 h using confocal microscopy with the Fluo-3-acetoxymethyl ester (Fluo-3AM) Ca^2+^ indicator (Figure S3B). Native S100A9 did not cause an increase in the free Ca^2+^ level after the addition to SH-SY5Y cells from 10 to 60 min, while both aged amyloid samples led to a similar increase of intracellular Ca^2+^, which was manifested in Fluo-3AM fluorescence (Figure S3B). Therefore, in further experiments, only the 48 h aged S100A9 samples were used with increasing OleA concentrations (Figure 7C). When the cells were treated with a 1:1 molar ratio of S100A9 to OleA, Fluo-3AM fluorescence subsided significantly. Remarkably, it returned to the level of untreated cells when SH-SY5Y cells were exposed to the samples with 1:5 and 1:10 of S100A9 to OleA molar ratios. This is consistent with the protective effect of OleA observed by MTT cell viability and the ROS assays presented above (Figure 7A,B).

### Effect of S100A9 Amyloids on Differentiated SH-SY5Y Neuroblastoma Cells

To compare the susceptibility of undifferentiated and differentiated SH-SY5Y cells to S100A9 amyloids, we repeated the MTT and ROS assays on differentiated SH-SY5Y cells. The cells were exposed for 24 h to 20 μM S100A9 fibrils (equivalent of the monomer concentration). The differentiated cells appeared to be slightly more susceptible to the amyloid induced damage in terms of both cell viability and ROS production (Figure S5). In agreement with our unpublished data, the different cell susceptibility could be due to a different poly- and monosialic acid expression on the plasma membrane. However, in this paper, we focused on the undifferentiated SH-SY5Y cells to compare our results with previously published studies.^11^

### OleA Interferes with S100A9 Amyloid Binding to Cell Membrane Rafts Studied by Immunofluorescence and Fluorescence Resonance Energy Transfer (FRET)

**Figure 8:**
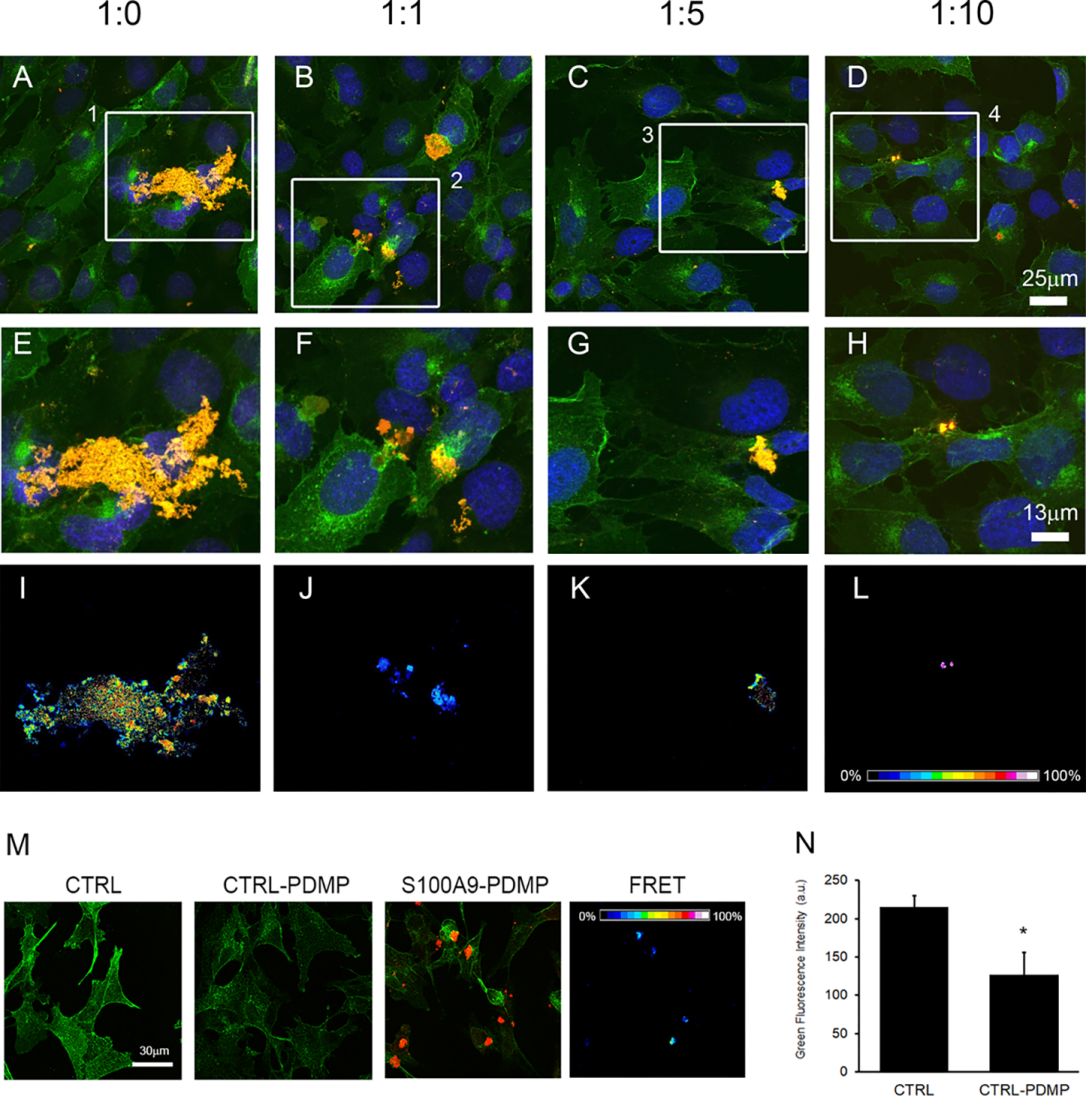
Immunolocalization of S100A9 aggregates on the SH-SY5Y plasma membrane. Confocal microscopy imaging of SH-SY5Y cells exposed for 24 h to 20 μM S100A9 aggregated for 48 h in the absence or in the presence of different molar ratios of S100A9 to OleA as indicated in the figures. The cell membranes were stained with Alexa 488-conjugated CTX-B (green fluorescence); cell nuclei were stained with Hoechst 33342 (blue fluorescence), and protein aggregates were stained with anti-S100A9 antibodies followed by treatment with Alexa 568-conjugated antirabbit secondary antibodies (red fluorescence). The mergence of the channels is shown in (A–D). Zoomed areas from (A–D) are shown in (E–H). FRET efficiency in those areas is shown in (I–L). (M) S100A9 do not bind to membrane rafts in the SH-SY5Y cells pretreated with an inhibitor of the GM1 synthesis as revealed by the lack of FRET efficiency. The control corresponds to the untreated cells. The SH-SY5Y cells were pretreated with 10 μM PDMD for 48 h (indicated as CTRL-PDMP) to reduce GM1 on the cell surface and then incubated for 24 h with 20 μM S100A9 fibrils (in monomer concentration). (N) Quantification of green fluorescence per cell, indicating the reduction of GM1 in cell membranes. Error bars indicate the standard error of three independent experiments. *p < 0.001 versus control.

The direct binding of amyloids to plasma membranes is a critical step in amyloid cytotoxicity.^46,47^ Notably, amyloids can accumulate in membrane rafts, containing monosialotetrahexosylganglioside-1 (GM1).^48^ The amyloid binding leads to structural and functional perturbations of the cell membrane, affecting signaling pathways^49−51^ and altering intracellular free Ca^2+^ and ROS levels.^44,52^ In addition, many papers have reported the effect on signaling pathways by direct binding of amyloids to cell membrane receptors.^52−55^ Therefore, we performed immunofluorescence assay with the GM1 and S100A9 specific fluorescently labeled antibodies using confocal microscopy and sensitized FRET analysis to evaluate the interactions between the membrane ganglioside GM1 and S100A9 amyloids produced after the 48 h incubation in the absence or presence of OleA. Confocal imaging revealed that the amyloid assemblies were accumulated at the SH-SY5Y plasma membrane and colocalized with GM1 rafts (Figure 8A,E). Moreover, a strong FRET signal indicates close spatial localization and even direct interactions of the S100A9 amyloids with the membrane GM1 (Figure 8I). When the cells were subjected to S100A9 amyloids developed at increasing OleA concentrations, we observed both smaller clusters of aggregates on the cell membrane (Figure 6F–H) and a reduced FRET signal (Figure 8J–L). Furthermore, to assess the direct involvement of ganglioside GM1 in the binding of S100A9 amyloids to the cell membrane, we also conducted immunofluorescence experiments after treating the cells with the ganglioside synthesis inhibitor d-threo-1-phenil-2-decanoylamino-3-morpholino-1-propanol (d-PDMD). We found that the binding of S100A9 fibrils to membrane rafts was significantly reduced in cells depleted of GM1, following their treatment with d-PDMD (Figure 8M,N). These data support the conclusion that the binding sites of S100A9 amyloids to the cell membrane occur at the raft domains. These observations also suggest the critical role of GM1 in binding of S100A9 amyloids to the cell membrane, most likely due to the clusters of sialic acid moieties, producing negatively charged regions at the cell surface. All results signify the roles of the amyloid–membrane interactions in triggering cellular toxicity and the importance of OleA in reducing cytotoxicity and perturbations of intracellular Ca^2+^ and ROS levels induced by S100A9 amyloids.

## Concluding Remarks

Here, for the first time, we have demonstrated that the most abundant polyphenol in olive oil, OleA, has multiple potencies in the inhibition of the amyloid self-assembly of pro-inflammatory protein S100A9 and mitigation of its damaging effect on neuroblastoma SH-SY5H cells. By directly interacting with native and fibrillar S100A9, OleA acts via multiple mechanisms and pathways to inhibit S100A9 amyloid aggregation; i.e., it prevents amyloid oligomerization and cross-β-sheet formation, reduces the effective rate of amyloid growth and the length of amyloid fibrils, and diminishes the overall amyloid load. Moreover, OleA is able to disintegrate already preformed fibrils of S100A9, converting them into nontoxic aggregates, which do not bind ThT dye, characteristic for cross-β-sheet amyloids. Furthermore, OleA targets S100A9 amyloids at the cellular membrane, protecting cells from amyloid cytotoxicity and amyloid-induced increased levels of ROS and free Ca^2+^. Since S100A9 is both a pro-inflammatory and amyloidogenic protein, OleA effectively mitigates the pathological consequences of the S100A9-driven amyloid-neuroinflammatory cascade in neurodegenerative diseases.^9−11^ Together with previously reported antiamyloid effects of OleA on other amyloidogenic polypeptides,^25−33^ the present findings further emphasize the beneficial properties of the Mediterranean diet in which olive oil is a central component.

## Methods

### Amyloid Fibril Formation

S100A9 protein was expressed in *E. coli* and subjected to purification procedures as reported previously.^56^ Lyophilized S100A9 was dissolved in PBS buffer at pH 7.4, with all solutions remaining on ice. In order to remove any aggregates, all S100A9 solutions were filtered through a 0.22 μm spin membrane filter prior to subjecting them to the amyloid incubation. To form amyloids, S100A9 was incubated in PBS, pH 7.4, at 42 °C. Previously, we have conducted extensive kinetics experiments on S100A9 amyloid formation at both 37 and 42 °C^19^ as well as at 55 °C.^57^ The scaling of the corresponding kinetics rate constants versus temperature illustrated that the mechanism of S100A9 amyloid self-assembly remained the same in this wide temperature range, which is below the thermal unfolding transition of S100A9, occurring above 60 °C.^56^ The morphology of amyloid fibrils, developed in this temperature range and followed by AFM, remained also the same.^19,57^ We selected the arbitrary condition at 42 °C in this research to speed up the amyloid self-assembly without altering the basic mechanism.

### Preparation of OleA

Oleuropein (extrasynthese) was deglycosylated by subjecting it to almond β-glucosidase treatment (EC 3.2.1.21, Fluka, Sigma-Aldrich) as specified previously.^27^ 10 mM oleuropein in 310 μL of 0.1 M sodium phosphate buffer, pH 7.0, was incubated with 8.9 IU of β-glucosidase overnight at room temperature (RT) and centrifuged at 18 000 rpm for 10 min. The pellet containing the deglycosylated OleA was resuspended in 100 mM stocks using DMSO, frozen, protected from light, and once opened, used within 24 h. The deglycosylation was verified by assaying in the supernatant the levels of glucose using the Glucose assay kit (HK, Sigma-Aldrich).

### Dissolving OleA

OleA was dissolved in 20% DMSO to reach a 50 mM concentration. A second dilution was made in 200 μL of PBS to reach a 5 mM concentration of OleA. Samples were sonicated during 2 min to dissolve the entire sample and manipulated at RT in the dark during the experiments. In the concentration-dependent experiments, OleA was subsequently diluted to 100, 200, 500, and 1000 μM. The same percentage of DMSO (0.4%) was kept in all solutions.

### ThT Fluorescence Assay

ThT is a fluorescence dye that binds to the β-sheet containing amyloid structures; this leads to an increase of its fluorescence and thus enables the kinetics of amyloid formation to be followed and quantified. The ThT assay was performed as described previously.^36^ 100 μM S100A9 was transferred into Corning 96 black well plates with transparent bottoms, and then, 20 μM ThT was added to every well. Sample volumes were kept at 200 μL per well. The 96 well plates were covered, transferred immediately into a Tecan F200 PRO plate reader, and subjected to incubation at 42 °C during 50 h by applying 432 rpm orbital shaking every 10 min. Fluorescence of ThT dye was recorded every 10 min. A filter at 430 nm was used for excitation, and a filter at 495 nm was used for emission; both filters were characterized by 20 nm band widths. All protein solutions were incubated in triplicates.

### AFM Imaging

AFM imaging was performed in air using a BioScope Catalyst AFM (Bruker) operating in a peak force mode. The scan rate was set at 0.51 Hz, and a scan resolution of 512 × 512 pixels was used. Bruker MSLN and SLN cantilevers were used in all measurements. Imaging was also conducted using a PicoPlus AFM (Molecular Imaging) equipped with a 100 μm scanner operating in tapping mode in air. The resonance frequency was set in the 170 to 190 kHz range; the scan rate was at 1 Hz, and the scan resolution was at 512 × 512 pixels. For ambient AFM imaging, 20 μL samples were deposited on freshly cleaved mica, kept for 30 min, washed 5 times with 200 μL deionized water, and left to dry at RT. The heights of the amyloid structures were measured in their AFM cross sections using Bruker Nanoscope AFM analysis software, and the amyloid fibril lengths were measured using ImageJ software.

### Dot Blot Analysis

S100A9 amyloid samples incubated with and without OleA were examined in dot blot experiments using amyloid oligomer specific antibodies.^58^ 2 μL of the amyloid sample was deposited on a nitrocellulose membrane (Fisher Thermo Scientific) and dried at RT for 15 min. Nonspecific antigen sites were blocked using 3% bovine serum albumin (BSA) in Tris buffer saline containing 0.05% Tween-20 (TBS-T) for 1.5 h at RT. Then, the nitrocellulose membrane was subjected to incubation with primary antibodies, i.e., rabbit A11 generic amyloid oligomer specific antibodies produced by Kayed et al.,^58^ and dissolved in 0.5% BSA in TBS-T for 30 min at RT. After that, the nitrocellulose membrane was washed 3 times with TBS-T for 5 min each wash and incubated with the secondary antibodies, i.e., antirabbit IgGs conjugated with horseradish peroxidase (AS10668 lot 1809 Agrisea), for 45 min at a 1:500 dilution at RT. The nitrocellulose membrane was washed 2 times with TBS-T and 1 time with TBS for 15 min each wash. After the addition of enhanced chemiluminescence reagent for 1 min, the membrane was subjected to the measurement of chemiluminescence by a Bio-Rad ChemiDoc Imaging System. The density of blots was measured using an ImageJ software.

### Titration of Native and Aggregated S100A9 by OleA Using Intrinsic Fluorescence

4 μM samples of either native or 24 h aged amyloid S100A9 were titrated by OleA in a 2 mm path length quartz cuvette using degassed PBS, pH 7.4, at RT. Intrinsic fluorescence spectra were acquired using a FP 6500 Jasco spectrofluorometer at RT. Excitation wavelength was set at 280 nm; fluorescence emission was recorded between 295 and 450 nm, and both excitation and emission slits were set at 3 nm. The spectra were obtained by averaging 3 scans recorded at a 200 nm/min rate. The OleA spectra at each added concentration were subtracted from those of S100A9.

### Titration of S100A9 Amyloids by OleA Monitored by ThT Fluorescence

150 μM S100A9 was aggregated for 48 h in degassed PBS, pH 7.4, at 42 °C. 20 μM ThT was added to each amyloid sample; then, they were diluted to a 75 μM S100A9 concentration. The titration by OleA was performed using 2 mm quartz cuvettes in a FP 6500 Jasco spectrofluorometer at RT. Excitation wavelength was set at 450 nm; fluorescence emission spectra were recorded between 480 and 550 nm, and both slits at excitation and emission were set at 10 nm. The spectra were obtained by averaging 3 scans recorded at a 200 nm/min rate.

### Fitting the Kinetics of S100A9 Amyloid Formation in the Absence and Presence of OleA

The S100A9 amyloid kinetics in the absence and presence of OleA were fitted using a nucleation dependent polymerization model.^38^ The initial parts (<20% plateau level) of the kinetic curves were fitted using the following equation:
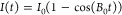
where *I*_0_ is the fluorescence intensity at the <20% plateau level and *B*_0_ is the effective rate constant, combining microscopic nucleation and growth constants.

### Fitting the Titration of S100A9 by OleA Monitored by Intrinsic Fluorescence

The maximum positions of the fluorescence spectra of S100A9, containing a single Trp 88 residue, were determined using a first derivative method. Prior to this, all spectra were smoothed by the Eilers smoothing algorithm, minimizing the noise level.^59^ The smoothing parameter λ was 10^5^, and the smoothing order was 3. The fluorescence intensity at the spectral maximum and the spectral maximum position errors were calculated by taking into account the instrument accuracy (Jasco FP6500), and 3 spectral replicates were recorded at each OleA concentration. The titration curve of S100A9 by OleA followed by fluorescence intensity at the spectral maximum was fitted by the two binding site model,^60^ relating four parameters:
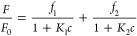
where *F* is the fluorescence intensity of S100A9 in the presence of each OleA concentration, *F*_0_ is the fluorescence intensity of S100A9 in the absence of OleA, *f*_1_ is the fractional occupancy of binding sites with affinity *K*_1_, and *f*_2_ is the fractional occupancy of binding sites with affinity *K*_2_.

### Far-UV CD

CD spectra were recorded using a Jasco J-810 spectropolarimeter. The measurements were performed in a quartz cuvette with 1 mm spectral pathway, and 3 repeats were averaged.

### MD

All atom MD calculations used the GROMACS 2019.3 program and protocols that were descried in our earlier studies.^37^

### Molecular Docking Studies

Possible binding sites for OleA on the S100A9 surface were at first explored by searching for hydrophobic patches and cavities on the protein Connolly surface.^61^ Docking positions were calculated using RxDock and AutoDock Vina 1.1.2.^62,63^ The ligands were hydrogenated and charged at pH 7.0 using the Gasteiger protocol. Proteins were protonated at pH 7.0 using the AMBER98S force field. The molecular structures for OleA were taken from ChemSpider and PubChem databases. The S100A9 structure was downloaded from the protein data bank, PDB: 5I8N.

### All-Atom Molecular Dynamics Studies

For MD computation, the S100A9-OleA complex was prepared using CHARMM-GUI solution builder.^64^ In our simulations, we used 85 080 water molecules (TIP3), 272 potassium ions (POT), and 221 chlorine ions (CLA). The simulation box was 112 × 112 × 112 Å^3^ in size and contained 254 871 atoms. The system was relaxed using a sequence of equilibration steps at 303.15 K using Nose-Hoover coupling, and the pressure was set to 1.0 bar using semi-isotropic Parinello-Rahman coupling. The constraint algorithm was LINCS, and the cutoff scheme was Verlet. Two minimization steps and one equilibration step were used for the system relaxation. The simulations analyzed molecular processes for 100 ns on a molecular time scale in 50 million steps with the step size set to 2 fs. The ligand parametrization was prepared using ACPYPE tools^65^ or the CHARMM-GUI Ligand Reader & Modeler for the calculation of CHARMM-compatible topology and parameter files.^66^ All simulations used GROMACS version 2019.4.^67^

### Neuroblastoma Cell Culture

Human neuroblastoma SH-SY5Y cells were grown in a 5.0% CO_2_ humidified atmosphere at 37 °C in 50% HAM, 50% DMEM, containing 10% fetal bovine serum (FBS), 3.0 mM glutamine, 100 units/mL penicillin, and 100 μg/mL streptomycin. The materials used for cell culture were from Sigma-Aldrich. To assess cell viability by the MTT reduction assay, neuroblastoma cells were grown in a 96 well plate at 15 × 10^3^ cells/well, while for immunofluorescence staining, they were cultured in a 24 well plate at 3 × 10^4^ cells/well. The nuclei of the cells were stained with 0.5 μg/mL Hoechst 33342 (Sigma-Aldrich) during a 30 min incubation at 37 °C; then, the cells were rinsed twice with PBS, and complete medium was added.

### Differentiating Neuroblastoma Cells

SH-SY5Y cell differentiation was induced by growing the cells for 7 days in culture medium supplemented with 3% FBS and 10 μM retinoic acid,^68^ and then, the cells were treated with S100A9 amyloids.

### MTT Viability Assay

The MTT cell metabolomic activity assay optimized for the SH-SY5Y cell line was performed to assess the cell viability. First, the neuroblastoma cell line was seeded and grown for 24 h in 96 well plates in complete medium. Then, the cells were treated for 24 h with 20 μM S100A9 in the native or aggregated state in the absence or presence of increasing OleA concentrations. After 24 h of coincubation of the cells with protein samples, the culture medium from each well was discarded and the cells were incubated for a further 1 h at 37 °C with 100 μL of serum free without phenol red DMEM containing 0.5 mg/mL 3-(4,5-dimethylthiazol-2-yl)-2,5-diphenyltetrazolium bromide (MTT) dye. Subsequently, cells were lysed by adding 100 μL of solubilizing solution (20% SDS, 50% *N*,*N*-dimethylformamide). After 2 h of lysis at 37 °C, the absorbance value of blue formazan, produced by viable cells, was determined by a spectrophotometric microplate reader at 570 nm. The average values were calculated from triplicate readings and subtracted from the blank value.

### Intracellular ROS Levels

Intracellular ROS levels were measured using fluorescent probe 2′,7′-dichlorofluorescein diacetate acetyl ester (CM-H_2_DCFDA, Thermo Fisher Scientific), which permeates the cell membrane and then becomes hydrolyzed and oxidized by radical species to fluorescent product, DCF. The formation of this product was monitored by the increase of fluorescence at 538 nm. SH-SY5Y cells were seeded in 96 well plates (3 × 10^4^ cells/well) and, after 24 h, were exposed during a further 24 h to 20 μM S100A9 in the native or aggregated form with or without increasing OleA concentrations. After that, DMEM, without phenol red and supplemented with 10 μM CM-H_2_DCFDA, was added to each well, and the samples were incubated for 30 min in the dark at 37 °C. The fluorescence at 538 nm was measured using a Fluoroscan Ascent FL (Thermo Fisher Scientific).

### Cytosolic Levels of Free Calcium Measured by Confocal Imaging

Fluorescent probe Fluo-3 acetoxymethyl ester (Fluo-3, Thermo Fisher Scientific) was used to detect intracellular levels of free Ca^2+^. Subconfluent neuroblastoma cells grown on glass coverslips were loaded with 5.0 μM Fluo-3 at 37 °C for 5 min and then treated for 10, 30, or 60 min with S100A9 aggregates formed after a 48 h incubation either without OleA or with OleA at different S100A9 to OleA molar ratios (1:1, 1:5, and 1:10). Finally, the cells were fixed for 10 min in 2.0% paraformaldehyde in PBS. Imaging was carried out using a confocal Leica TCS SP8 scanning microscope with a Leica Plan 7 Apo 40× oil immersion objective.

### Immunofluorescence Confocal Imaging of S100A9 Amyloid Interactions with Cell Membranes

Subconfluent neuroblastoma cells were seeded on glass coverslips and exposed for 24 h to 2.0 μM S100A9 amyloids incubated for 48 h either without OleA or with OleA at different S100A9 to OleA molar ratios (1:1, 1:5, and 1:10). After cell washing with PBS, gangliosides GM1 in live cell plasma membranes were labeled with 10 ng/mL cholera toxin B-subunit (CTX-B) conjugated with Alexa 488 (Thermo Fisher Scientific) in fresh complete medium for 10 min at RT. Cells were subsequently fixed by 2.0% paraformaldehyde in PBS for 10 min and treated with a 1:1 acetone/ethanol solution for 4.0 min at RT to allow cell permeabilization. After thorough PBS washing, the blocking was performed using PBS containing 0.5% BSA and 0.2% gelatin for 1 h at 37 °C. S100A9 in the neuroblastoma cell membranes was stained for 1.0 h at RT with rabbit anticalgranulin B (B-5) monoclonal antibody (sc-376772, Santa-Cruz Biotechnology) diluted to 1:300 in the blocking solution. Then, the cells were washed 3 times with PBS for 30 min under stirring, and then, Alexa 568 conjugated antirabbit secondary antibodies (Thermo Fisher Scientific) diluted to 1:100 in PBS were added for 30 min at RT. Finally, the samples were washed twice in PBS and once in distilled water, and the coverslips were mounted. Fluorescence imaging was performed using a Leica TCS SP8 AOBS confocal scanning microscope. Cell imaging was acquired in at least two different experiments using a Leica HC PL Apo CS2 63× oil immersion objective. Spectral analysis of the FRET interaction between Alexa 488 fluorophore conjugated with CTX-B and Alexa 568 fluorophore on immunolabeled S100A9 was carried out by the FRET sensitized emission method, as previously reported.^32,69^

### Statistical Analysis

One-way analysis of variance by ANOVA and pairwise comparisons by the Tukey HSD (honestly significant difference) method were used for the statistical evaluation of the data.

## Supporting Information Available

Time dependences of ThT fluorescence during OleA incubation; interaction of S100A9 amyloids with A11 amyloid oligomer specific antibodies upon increasing OleA concentration; cell viability and intracellular free Ca^2+^ level in SH-SY5Y cells affected by S100A9 amyloids; preformed S100A9 fibrils treated with OleA during various times; cytotoxicity of S100A9 fibrils to undifferentiated and differentiated SH-SY5Y cells (PDF)

## Abbreviations

AD: Alzheimer’s disease
AFM: atomic force miscroscopy
CD: circular dichroism
FRET: fluorescence resonance energy transfer
MD: molecular dynamics
OleA: oleuropein aglycone
PD: Parkinson’s disease
ROS: reactive oxidative species
RMSD: root-mean-square deviation
ThT: thioflavin-T
