# Supplementary material for: Natural Compound from Olive Oil Inhibits S100A9 Amyloid Formation and Cytotoxicity: Implications for Preventing Alzheimer’s Disease
Source: ACS Chem Neurosci. 2021 May 12;12(11):1905–18. doi: 10.1021/acschemneuro.0c00828 (PMC8291483; doi:10.1021/acschemneuro.0c00828)
Supplement: Supplementary file 1 — cn0c00828_si_001.pdf [file cn0c00828_si_001.pdf]

Supporting Information

NATURAL COMPOUND FROM OLIVE OIL INHIBITS S100A9 AMYLOID FORMATION AND CYTOTOXICITY: IMPLICATIONS FOR PREVENTING ALZHEIMER'S DISEASE

Manuela Leri,[‡] Himanshu Chaudhary,[‡] Igor A. Iashchishyn,[‡] Jonathan Pansieri,[‡] Željko M. Svedružić,[‡] Silvia Gómez Alcalde, Greta Musteikyte, Vytautas Smirnovas, Massimo Stefani, Monica Bucciantini* and Ludmilla A. Morozova-Roche*

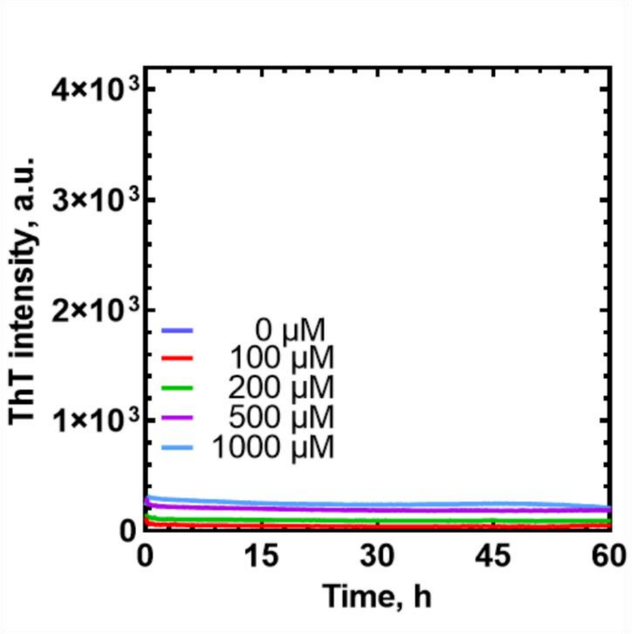

Figure S1. **Time dependences of ThT fluorescence during incubation of various concentrations of OleA.** Each assay was made in triplicates. PBS, pH7.4 and 42 °C.

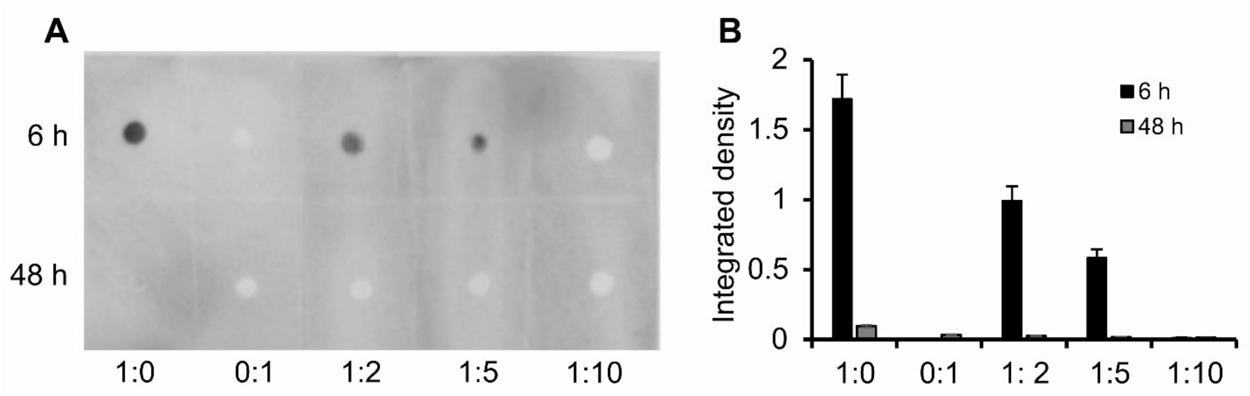

Figure S2. Interaction of S100A9 amyloids with A11 amyloid oligomer specific antibodies upon increasing OleA concentration. (A) Dot blots of S100A9 amyloids after 6 h and 48 h incubation in the absence and presence of increasing OleA concentration with A11 oligomer-specific antibodies. (B) Integrated density of dot blots versus increasing OleA concentrations. Molar ratio of S100A9 to OleA are as following: 1:0, 0:1, 1:2, 1:5 and 1:10 as indicated in caption.100 µM S100A9, PBS, pH 7.4 and 42 °C.

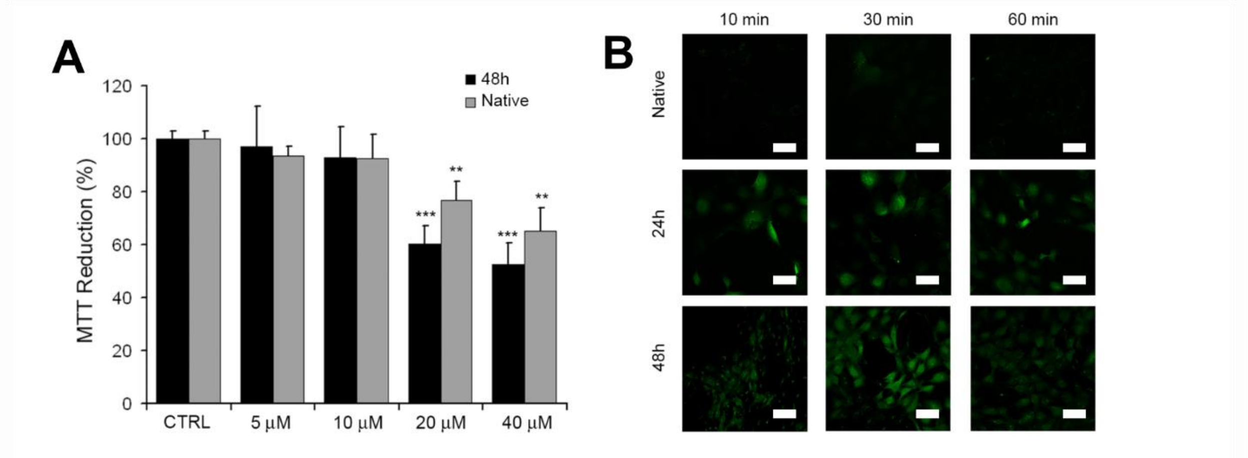

Figure S3. Cell viability and intracellular free Ca^{2+} level in SH-SY5Y cells affected by S100A9 amyloids. (A) Viability of SH-SY5Y cells assessed by MTT assay. Cells were treated for 24 h with different concentrations of native S100A9 and S100A9 fibrils incubated for 48 h in PBS, pH 7.4 and 42 °C. Error bars indicate the standard error of the mean of independent experiments carried out in triplicate. ***$p<0.001$ and **$p<0.01$ *versus* control. (B) Confocal microscopy imaging of intracellular free Ca^{2+} levels in SH-SY5Y cells exposed for 10, 30 and 60 min to 20 µM S100A9 in native or amyloid forms, the latter were incubated for 24 and 48 h in PBS, pH 7.4 and 42 °C, respectively.

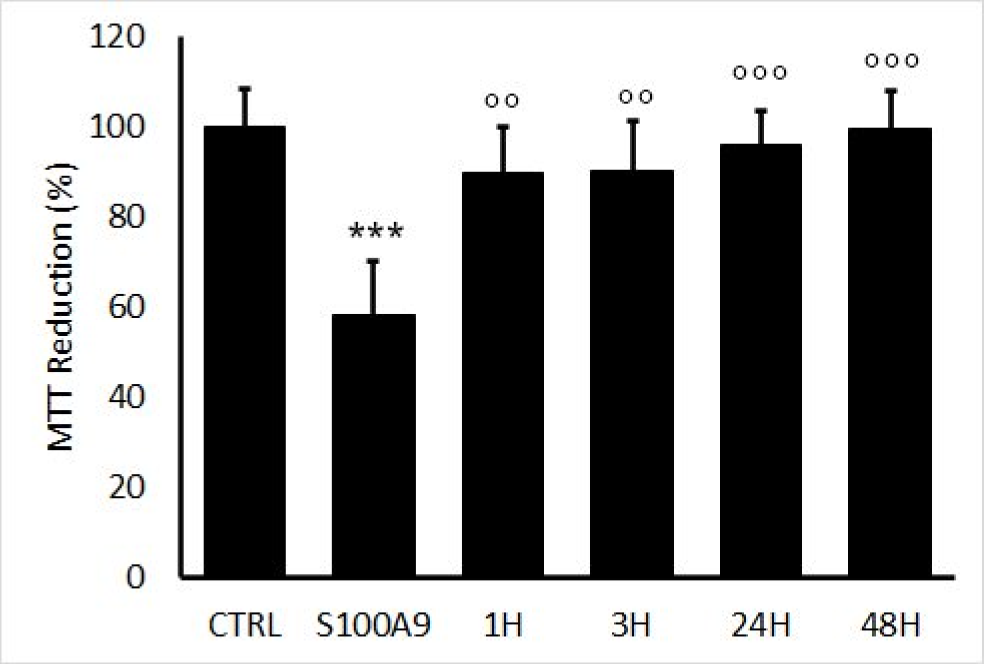

Figure S4. Preformed S100A9 fibrils treated with OleA during various times became non-toxic to SH-SY5Y cells. Preformed fibrils of S100A9 were treated with OleA at a 1:2 molar ratio (S100A9 : OleA) during increasing times and tested on SH-SY5Y cells. Cytotoxicity was assessed by MTT assay after 24 h of cell exposure to the S100A9 samples. Time of treatment of S100A9 amyloids by OleA is shown along *x*-axis; S100A9 sample alone was not treated with OleA. Control corresponds to untreated cells. Error bars indicate the standard error of three independent experiments carried out in triplicate. *** $p<0.001$ *versus* control. °° $p<0.05$; °°°$p<0.01$ *versus* S100A9 fibrils. Preformed fibrils were assembled during 48 h incubation of 75 µM S100A9 in PBS, pH 7.4 and 42 °C.

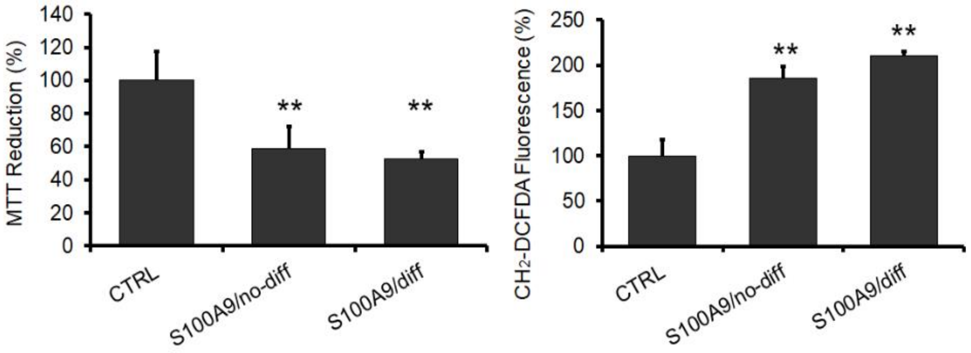

Figure S5. Cytotoxicity of S100A9 fibrils to undifferentiated and differentiated SH-SY5Y cells. The cells were differentiated with retinoic acid and were exposed to S100A9 fibrils (20 μM) for 24 h. Cytotoxicity was assessed by the MTT (left) and ROS (right) assays. Control corresponds to untreated cells. Error bars indicate the standard error of three independent experiments carried out in triplicate. ** $p<0.01$ *versus* control.
